# Primary mucinous adenocarcinoma of the orbit: A rare clinical entity

**DOI:** 10.1002/ccr3.5472

**Published:** 2022-02-16

**Authors:** Kais Maamri, Rihab Ben Fredj, Nesrine Nessib, Amine Trifa, Maher Hadhri, Ghassen Elkahla, Atef Ben Nsir, Mehdi Darmoul

**Affiliations:** ^1^ Department of Neurosurgery Fattouma Bourguiba University Hospital of Monastir Monastir Tunisia

**Keywords:** enucleation, exophthalmos, mucinous adenocarcinoma, orbital tumor

## Abstract

Primary mucinous adenocarcinoma is an exceptionally rare neoplasm with a propensity for local recurrence and metastasis. We report the second case in the world literature of a primary mucinous adenocarcinoma of the orbit in a 66‐year‐old man suffering from pain, progressive protrusion of left eye, and a deep drop in vision on the left for several weeks. His first external examination revealed significant proptosis with downward displacement of the left globe with no signs of lagophthalmos. A limitation of abduction was also noted. A CT of the orbit with and without contrast showed intra‐ and extra‐conical solid expansive process. MRI of the orbit with contrast and without contrast has shown a process of the supero‐internal angle of the left orbit. The patient was operated via a combined approach, and complete enucleation was done. The final pathologic diagnosis was mucinous adenocarcinoma of the orbit. The postoperative neuroimaging showed a complete resection of the tumor. The patient is referred for adjuvant radiotherapy. A CT of the orbit was made 3 months postoperatively and did not show any local recurrence.

## Graphical abstract

Mucinous adenocarcinoma is challenging to distinguish whether it is a primary neoplasm or an orbital metastasis of adenocarcinoma. Extensive metastatic workup for any primary malignancy elsewhere should be done. No matter it is a mucinous adenocarcinoma, or any other tumor growing in this region, the surgical approach is the same.

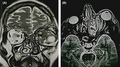

## 1 INTRODUCTION

The most common malignant tumor of eyelid and surrounding structures are basal cell carcinoma (BCC) 80%–90%, followed by squamous‐cell carcinoma ‐ 40%, sebaceous carcinoma ‐ 5%, and melanoma 1%.^1^ Primary mucinous adenocarcinoma is an exceptionally rare neoplasm with a propensity for local recurrence and metastasis, which may take its origins in the breast, gastrointestinal tract, thyroid, and glands of the skin. There is a strong inclination that rare mucinous adenocarcinoma emerging from the skin has a propensity for the eyelid.^2^ While eyelid neoplasm has been widely reported in the ophthalmology literature, the intraconic localization has not been described. Only one case of infiltrating adenocarcinoma with some features suggestive of mucoepidermoid carcinoma of the orbit has been reported in the literature.^3^ We report the case of a primary mucinous adenocarcinoma of the orbit in a 66‐year‐old man with its clinical, histological, and management features.

## 2 CASE REPORT

A 66‐year‐old man without significant medical history was admitted to our Neurosurgery Department suffering from pain, progressive protrusion of left eye, and a deep drop in vision on the left for several weeks. There is neither diplopia nor periorbital swelling. There were no other systemic complaints. First external examination revealed significant proptosis with downward displacement of the left globe with no signs of lagophthalmos. A limitation of abduction was also noted. Visual acuity without correction was normal in the right eye whereas in the left one there was no perception of light. No papillary edema was revealed. Intraocular pressures were 18 mm Hg in the right and 16 mm Hg in the left. Routine blood investigations were normal.

**FIGURE 1:**
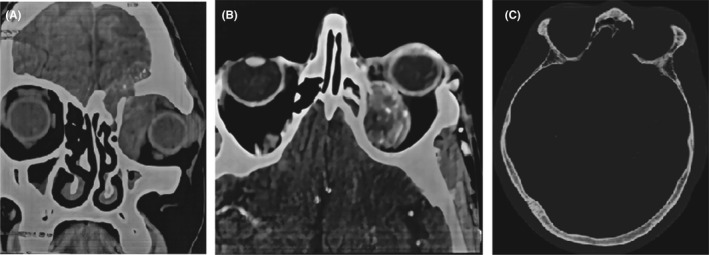
CT orbit coronal cut without contrast (A), axial cut with contrast (B), and axial cut bone window (C) demonstrating the large orbital mass surrounding and indenting the left globe with osteolysis

A CT of the orbit with and without contrast showed intra‐ and extra‐conical solid expansive process. It is centered on the internal rectus muscle and has a large implantation base. The lesion pushes the eyeball back and forth, and it is responsible for exophthalmos with osteolysis (Figure 1).

**FIGURE 2:**
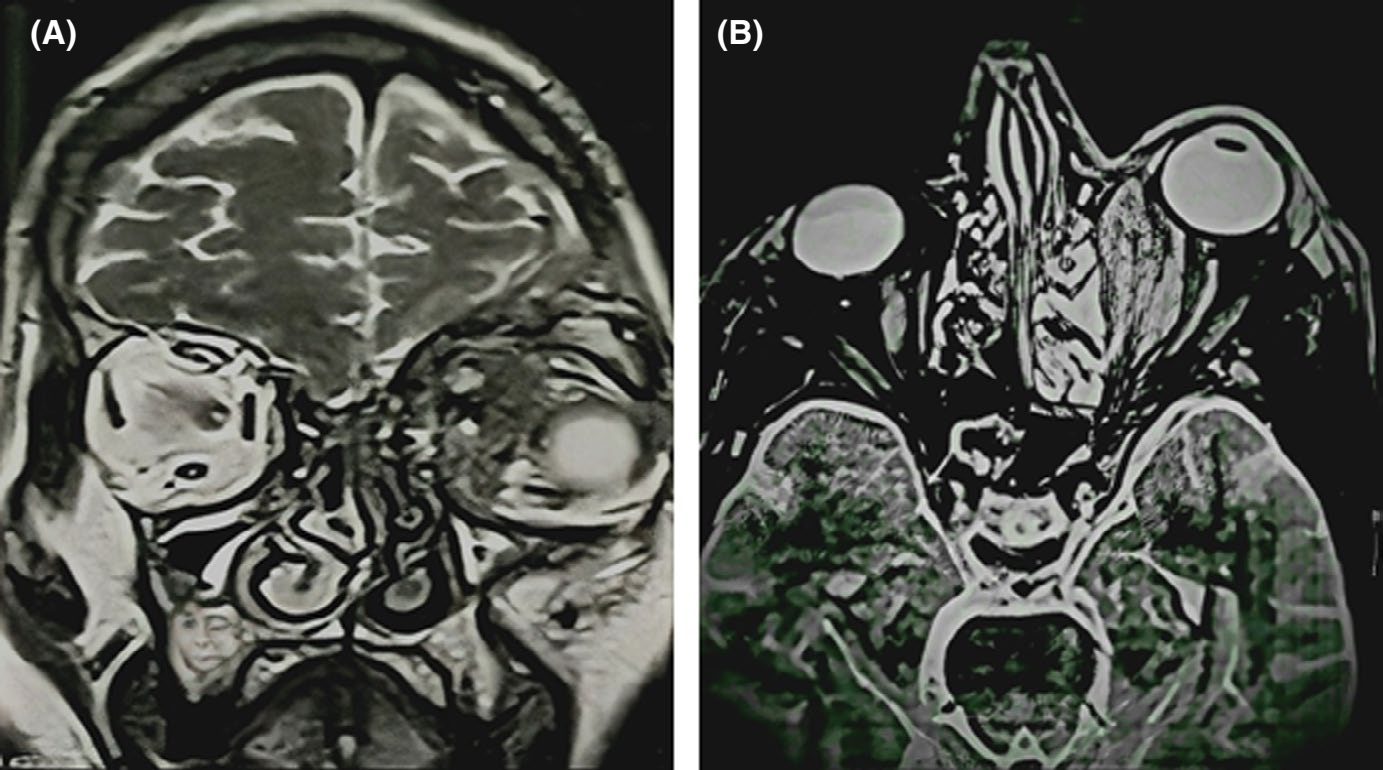
Brain magnetic resonance imaging (MRI) coronal T2‐WI (A) and axial T2‐WI (B) showing the large orbital mass surrounding and indenting the left globe

An MRI of the orbit with contrast and without contrast has shown a process of the supero‐internal angle of the left orbit, well limited, with an important enhancement after injection. This lesion is both intra‐ and extra‐conal, exerting a mass effect on the internal rectus muscle and the optic nerve (Figure 2).

Our patient had shown negative metastatic workup for any primary malignancy elsewhere. We completed with a thoraco‐abdomino‐pelvic CT scan and a colonoscopy, which returned normal.

**FIGURE 3:**
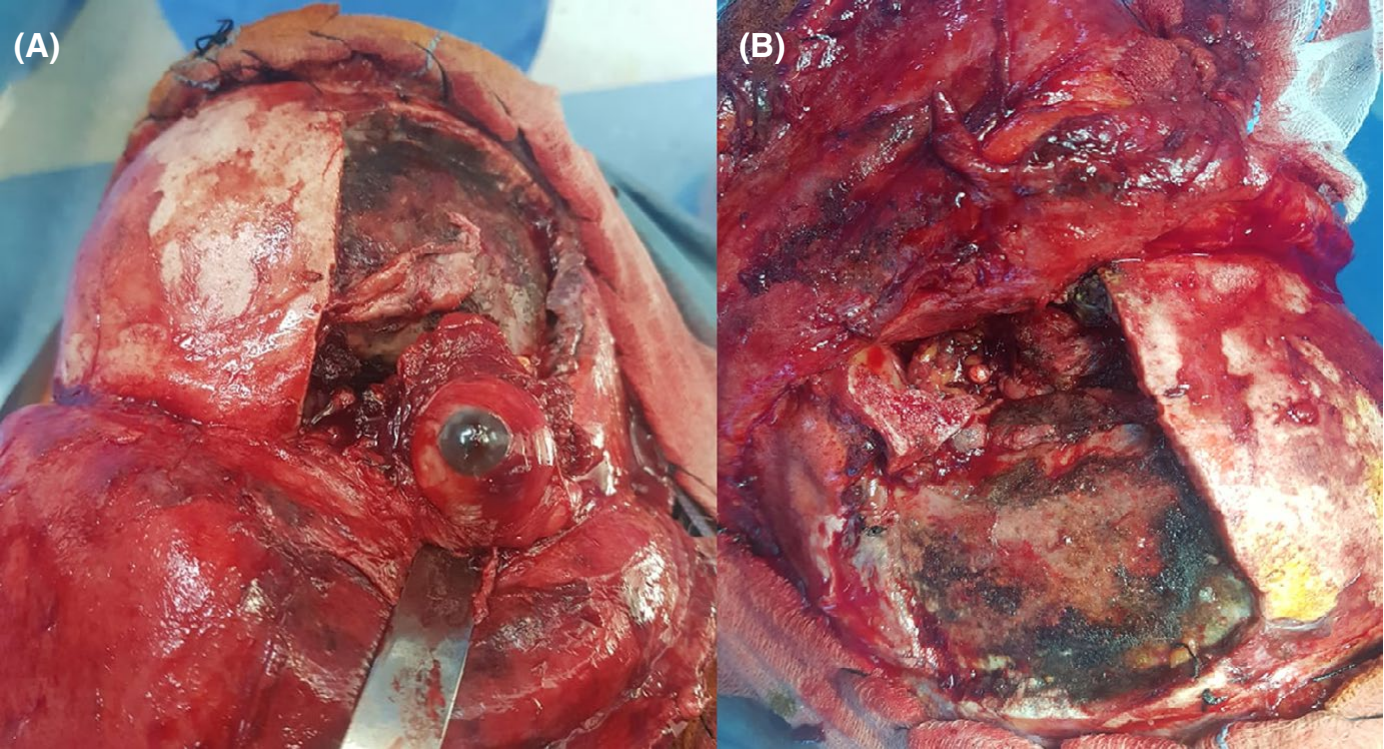
Intra‐operative view of the fronto‐orbito‐zygomatic approach, the excision of the orbital mass, and complete enucleation

**FIGURE 4:**
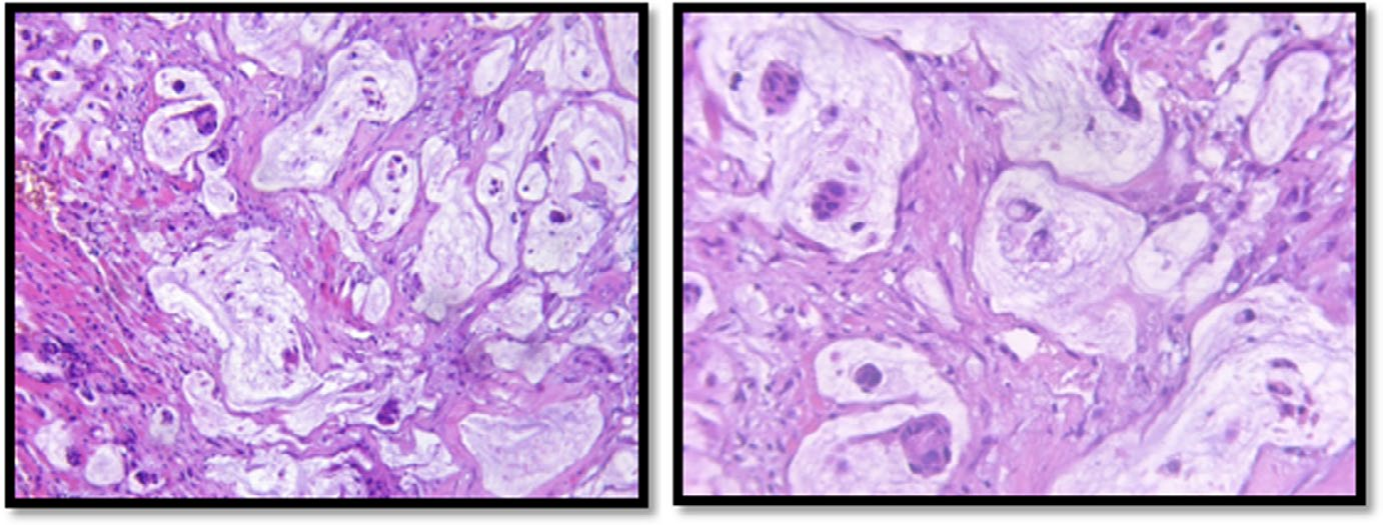
Carcinomatous proliferation made by patches of mucus in which tumor cells are bathed, often isolated or arranged in small clusters. These cells are of mucosecreting ring‐like appearance with an eccentric nucleus, mild‐to‐moderate nuclear atypia, and some mitosis. The complementary immunohistochemical study showed an intense cytoplasmic staining of 20% of the tumor cells with the anti‐CK7 and an intense and cytoplasmic staining of rare cells (less than 20%) with the anti‐CK20

The patient was operated via a combined approach. Through a fronto‐orbito‐zygomatic approach, excision of a firm hard lesion infiltrating the eyeball as well as the optic nerve, which motivated complete enucleation (Figure 3). We ended with a tarsorrhaphy. A total exenteration was ultimately performed while surgical specimens were sent for histopathologic evaluation. The final pathologic diagnosis was mucinous adenocarcinoma of the orbit (Figure 4).

**FIGURE 5:**
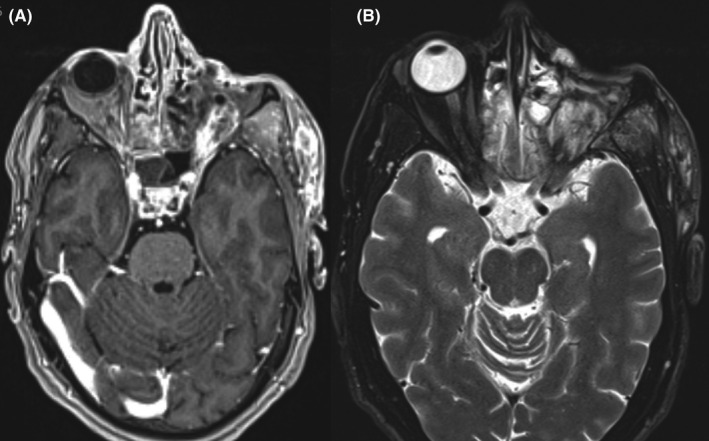
Brain magnetic resonance imaging (MRI) axial T1‐WI with gadolinium (A) and axial T2‐WI (B) showing the locoregional recurrence of the left orbital mass

Postoperative course was uneventful: No eyeball movement disorders or vision changes on the right side were noted, and the patient did not have any mucus leakage to the orbit or the nasal cavity. Postoperative neuroimaging showed a complete resection of the tumor. The patient is referred for adjuvant radiotherapy. Eight‐month follow‐up imaging showed locoregional recurrence of the orbital mass (Figure 5).

## 3 DISCUSSION

Primary mucinous adenocarcinoma is uncommon, with no predisposing factors or etiology.^2^ It can arise throughout the body especially in the head and in the neck. Mucinous adenocarcinomas emerging from the skin has a propensity for the eyelid. The rate of occurrence of this tumor in the eyelid varied from 30 to 45% according to several studies.^4^ The average age of patients with primary mucinous adenocarcinoma varied between the sixth and seventh decades.^4^ The age of our case study patient was 66 years old, which is compatible with the previous studies.

The problem here is to distinguish whether it is a primary mucinous adenocarcinoma of the orbit or is it just an orbital metastasis of a known adenocarcinoma. That is why many investigations should be done to determine the origin of this neoplasm. It is also worth noting that metastatic lesions involving the orbit were found to be rare, comprising between 1% and 13% of all orbital tumors.^2^ Orbital metastases are a rare manifestation of systemic malignancy, which classically occurs late in the evolution of a known neoplasm. However, in 42%–61% of cases, the orbital symptoms preceded the detection of the primary tumor.

Prompt surgical intervention with biopsy enabled definitive diagnosis of mucinous adenocarcinoma, leading to the discovery of the primitive tumor.^5^ So far, there have been only 6 known cases of metastatic mucinous adenocarcinoma in eye orbit. Five arose from gastrointestinal primaries including rectal, esophageal, gastric, and pancreatic cancer. Meanwhile, the sixth represented orbital reoccurrence from a primary eyelid sweat gland mucinous adenocarcinoma.^5^, ^6^, ^7^, ^8^, ^9^

At times, ocular symptoms have preceded the diagnosis of the primary lesion. And this highlights the importance of a thorough investigation aiming to search for the primary neoplastic process.^2^ In medical literature, there is only one case of primary infiltrating adenocarcinoma of the orbit, with some features suggestive of carcinoma initially diagnosed as idiopathic sclerosing orbital inflammation. The tumor did not appear to be cytologically aggressive, but rather had an infiltrative growth pattern suggestive of a high‐grade tumor, T4N0M0.

As demonstrated in that case, even though the biopsy concluded in benign lesion, the clinician must remain vigilant if he suspects signs of malignancy. This is particularly true in the presence of atypical presentation or signs of severity.^3^

Regardless of the orbital mass nature, clinically these tumors usually present symptoms such as proptosis, which is due to the infiltration of fat and extra‐ocular muscles by the tumor. Muscle involvement has been noted to produce diplopia. Pain was generally associated with periosteal and bone involvement. Pulsations were due to bone destruction or vascular tumors. Ptosis, a palpable mass, enophthalmos, and decreased vision had also been also observed.^10^

There are no radiological specificities in this type of tumor compared with other orbital masses. The MRI of the orbit with and without contrast may show a heterogenous orbital process, well limited, or infiltrating, with an important enhancement after injection. This lesion can be both intra‐ and extra‐conal and can exert a mass effect on rectus muscles and the optic nerve.

A careful histopathological examination must be carried out for a definitive diagnosis of a mucinous adenocarcinoma. On cross‐examination, it showed a white and firm unencapsulated tumor mostly fixed to the dermis.^4^

It is worth mentioning that histologically, it may be very awkward to distinguish metastatic mucinous adenocarcinoma from primary entities. Orbit and eye mucinous adenocarcinoma closely resemble those of the ovary, breast, rectus, bronchus, and colon adenocarcinomas. Characteristics round cuboidal cells with pale sialomucin‐rich cytoplasm vacuoles, surrounded by extracellular mucin pools, positive for EMA, PAS, alcian blue, as well as mucicarmine histochemical staining, are seen in both primary and metastatic lesions. The latter may show greater mitotic activity and pleomorphism on light microscopy. Stem cell origin studies will also be helpful to differentiate metastatic mucinous adenocarcinomas from primary one.^2^

The mucin of PMA is positive for periodic acid‐Schiff (PAS), alcian blue, mucicarmine, aldehyde fuchsin, and colloidal iron stains. The mucin is diastase and hyaluronidase resistant sialidase labile, thus making it nonsulfated sialomucin and distinguishable from hyaluronidase‐sensitive sulfomucin from other sweat gland tumors, basal cell carcinoma, lacrimal gland tumors, and metastatic mucinous adenocarcinoma of the gastrointestinal tract.

The immunohistochemistry has greatly helped in differentiating primary mucinous adenocarcinomas from metastatic entities. The expression pattern of a CK panel is very helpful. The primary one is CK7 positive and CK20 negative, whereas metastatic adenocarcinoma from the gastrointestinal tract is CK7 negative but CK20 positive. Some other helpful immunohistochemicals such as the carcinoembryonic antigen, the epithelial membrane antigen, and CK AE1/E3 may be helpful markers, which will point its origin from a secretory lobule. Gross cystic disease fluid protein‐15 (GCDFP‐15), estrogen receptor (ER), and progesterone receptor (PR) are also useful markers to differentiate primary mucinous adenocarcinoma from a metastatic breast adenocarcinoma. Cytokeratin 5/6 and p63 were the recently added markers and which have proven to be also efficient.^4^, ^11^

In our case, the histological examination has showed carcinomatous proliferation made by patches of mucus in which tumor cells are bathed, often isolated or arranged in small clusters. These cells are of mucosecreting ring‐like appearance with an eccentric nucleus, mild‐to‐moderate nuclear atypia, and some mitosis. The complementary immunohistochemical study showed an intense cytoplasmic staining of 20% of the tumor cells with the anti‐CK7 and CK20 negative.

For the first case of primary mucinous adenocarcinoma of the orbit described in the literature, additional surgery was suggested after biopsy, whether to be implemented with or without radiation and chemotherapy. The patient ultimately decided to undergo adjuvant radiation therapy. Neuroimaging after six months of post‐exenteration showed no signs of recurrence.^3^ The patient in our case received adjuvant radiotherapy after complete exenteration. A CT of the orbit was made 3 months postoperatively and did not show any local recurrence.

## 4 CONCLUSION

Primary mucinous adenocarcinoma of the orbit is an extremely rare neoplasm. Our case is the second case described in literature. A few cases of metastatic mucinous adenocarcinoma in the orbit have been described. It is worthwhile noting that histological and immunohistochemical features have been extremely helpful in the diagnosis of the primary etiology, but they surely could not exclude the metastatic etiology. Primary mucinous adenocarcinoma is locally aggressive with a recurrence rate up to 40%, that is why the mainstay of its treatment is a large local excision. The formerly cited entities have been extremely difficult to diagnose. A workup must be done to look for a primary lesion in all patients with identified mucinous adenocarcinoma.

## CONFLICT OF INTEREST

The authors declare no conflict of interest.

## AUTHOR CONTRIBUTIONS

Kais Maamri treated the patient and wrote the manuscript. Rihab Ben Fredj performed the acquisition, analysis, and interpretation of data with support from Nesrine Nessib and Amine Trifa. Maher Hadhri did the conception and the design of the project. Ghassen Belkahla prepared pathology figures and slides. Atef Ben Ncir and Mehdi Darmoul helped supervising the project. All authors discussed the results and contributed to the final manuscript.

## ETHICAL APPROVAL

The patient and his family consented to participate and publish their clinical data.

## CONSENT

Written informed consent was obtained from the patient to publish this report in accordance with the journal's patient consent policy.

## Data Availability

Data sharing not applicable to this article as no datasets were generated or analysed during the current study.
